# Malnutrition in maintenance hemodialysis: Prevalence, patterns, and clinical implications in Bangladesh

**DOI:** 10.1371/journal.pone.0354173

**Published:** 2026-07-17

**Authors:** Tanjina Rahman, Nasrin Sultana, Shakil Ahmed, Farhana Akter, Marium Sultana, Md Sajjadul Haque Ripon, Harun-ur Rashid

**Affiliations:** 1 Institute of Nutrition and Food Science, University of Dhaka, Dhaka, Bangladesh; 2 Department of Food Technology and Nutrition Science, Noakhali Science and Technology University, Noakhali, Bangladesh; 3 Department of Nutrition and Food Engineering, Daffodil International University, Dhaka, Bangladesh; 4 Department of Nephrology, Kidney Foundation Hospital and Research Institute, Dhaka, Bangladesh; Japanese Red Cross Medical Center, JAPAN

## Abstract

Malnutrition is a major clinical challenge among individuals receiving maintenance hemodialysis (HD), contributing to increased complications and reduced survival. The burden is particularly pronounced in resource-limited settings, where gaps in nutritional assessment and management persist. This cross-sectional study evaluated the prevalence and determinants of malnutrition among 181 HD patients from four districts in Bangladesh. Based on the International Society of Renal Nutrition and Metabolism (ISRNM) criteria, 42.5% of patients were malnourished, including 10.5% with severe malnutrition, while the Malnutrition Inflammation Score (MIS) identified malnutrition in 72.9% of participants. Patients who reused dialyzers more than twice had significantly higher odds of malnutrition [AOR = 5.8; 95% CI: 1.2–28.7]. Age, body mass index (BMI), and diabetes mellitus were also independently associated with poor nutritional status. These findings underscore the need for strengthened nutritional assessment and early identification of at-risk patients in HD care. Implementing validated diagnostic tools, such as those used in this study, may help reduce disease burden and improve survival outcomes in resource-constrained dialysis settings.

## Introduction

Chronic kidney disease (CKD) remains a major global health challenge, with its prevalence rising most sharply in resource-limited regions. Countries such as Bangladesh face substantial barriers to early detection and management due to limited screening practices and the high cost of renal care. CKD is diagnosed by a persistent decline in renal function-commonly indicated by markers of kidney damage or an estimated glomerular filtration rate (eGFR) below 60 ml/min/1.73 m^2^ [1]. Its pathogenesis involves a wide constellation of risk factors, including diabetes, hypertension, smoking, obesity, ageing, hypercholesterolemia, family history, inadequate hydration, medication effects, and exposure to environmental toxins, reflecting the complex biological and environmental interactions that shape CKD progression [2,3].

As CKD advances, many patients ultimately progress to End-Stage Renal Disease (ESRD), requiring renal replacement therapy to sustain life. Hemodialysis (HD) is the predominant modality in this context and serves as a critical support for patients with ESRD [4,5]. However, despite its life-saving role, HD itself may intensify metabolic and nutritional stress. Dialysis sessions are known to increase energy expenditure [6], while hormonal disturbances [7], growth hormone and insulin resistance [8], chronic inflammation [9], and thyroid dysfunction [10], further compound nutritional instability.

Within this landscape, protein-energy wasting (PEW) has emerged as a particularly concerning complication. PEW-characterized by inadequate nutrient intake relative to metabolic demands-is common among HD patients and is strongly associated with poor clinical outcomes, including increased hospitalization and mortality [11,12]. Reduced appetite, medication side effects, and stringent dietary restrictions contribute to this vulnerability [13], while the HD process itself increases the risk of malnutrition [14]. The burden of anorexia in HD patients stems from multiple influences, including uremic toxicity, chronic inflammation, depression, challenges with food access and preparation, reduced quality of life, and the catabolic consequences of dialysis techniques [15,16].

Globally, the prevalence of CKD continues to rise at an estimated rate of 7% per year, with the majority of cases occurring in adults [17,18]. In Bangladesh, the situation is particularly concerning: CKD affects an estimated 22.48% of the population-exceeding global averages [19]. Across Asia, CKD prevalence ranges from 10% to 18%, reflecting a widespread regional health burden. Likewise, CKD-related malnutrition remains a major challenge worldwide, with prevalence estimates ranging from 20% to 75% among hemodialysis patients, depending on diagnostic criteria and study population [15,20–22].

Dietitians play a central role in identifying and managing PEW and malnutrition in individuals receiving HD [23]. Comprehensive evaluation typically involves anthropometry, biochemical testing, dietary assessment, and tools such as the Subjective Global Assessment (SGA) [24]. Yet, despite the well-established importance of nutrition in HD care, many dialysis units fall short in routine nutritional monitoring [25]. In Bangladesh, the lack of a formal training pathway for renal dietitians further limits consistent and specialized nutrition care for this vulnerable population [26].

Given these gaps, this study sought to assess the prevalence of malnutrition among maintenance HD patients in Bangladesh using validated diagnostic approaches, including the International Society of Renal Nutrition and Metabolism (ISRNM) criteria and the Malnutrition Inflammation Score (MIS). By integrating anthropometric, dietary, clinical, and biochemical indicators, the study aims to clarify the factors contributing to nutritional deterioration in this setting. The findings are expected to support improvements in nutritional screening and patient management, particularly within the constraints of healthcare systems in developing countries.

## Methodology

### Study design and sample size

This cross-sectional study was conducted across four districts in Bangladesh-Dhaka, Mymensingh, Cumilla, and Noakhali between December 2021 and May 2022 using a pre-tested questionnaire to determine the prevalence of malnutrition among maintenance HD patients. The tool captured sociodemographic characteristics, medical history, HD-related information, laboratory values, clinical signs, dietary intake, and anthropometric parameters. The initial sample size of 267 was calculated using a 95% confidence interval (CI), a 5% margin of error, and a CKD prevalence of 22.48% in Bangladesh [19], applying the formula [27]:


$$\begin{array}{c} n=\frac{Z^{\text{2}}\times P(\text{1}-P)}{d^{\text{2}}} \end{array}$$

Where *Z* = Standard Deviation = 1.96 (95% CI), n = Sample size, P = Prevalence of CKD, *P* = 0.2248, [19] and *d* = Margin of error = 0.05.

Participants were recruited using non- probability convenience sampling from selected dialysis centers. As a non-probability sampling method, convenience sampling may have introduced selection bias, as patients attending the selected facilities and those willing to participate may differ systematically from the broader population of maintenance hemodialysis patients in Bangladesh. Consequently, the findings should be generalized with caution. However, due to the limited number of eligible maintenance hemodialysis patients available at participating facilities during the study period and the application of predefined inclusion and exclusion criteria, 181 patients were ultimately recruited and included in the final analysis. Eligible participants were ≥18 years old, on HD for more than three months, medically stable with a life expectancy >1 year, and able to provide informed consent. Exclusion criteria included major surgery within the previous six months, planned living-donor transplantation, active treatment for cancer or HIV/AIDS, pregnancy, lactation, or intention to conceive, ensuring a homogeneous study cohort.

### Anthropometric measurements

All anthropometric measurements were performed by trained investigators following standardized protocols. Height was measured to the nearest 0.1 cm using a stadiometer, and weight was assessed using a digital scale (TANITA HD 313, USA). Pre- and post-dialysis weights were obtained following standard procedures [28]. Body mass index (BMI) was calculated as post-dialysis weight divided by height squared (kg/m^2^), and categorized based on WHO cutoffs [29]. Following National Kidney Foundation (NKF) guidelines, BMI < 23 kg/m^2^ was considered a marker of increased morbidity and mortality risk in HD patients [30].

Dry weight, triceps skin-fold thickness (TSF), and mid-arm circumference (MAC) were obtained 10–20 minutes after dialysis, measured on the non-access arm. TSF was measured using a Harpenden caliper (HSK-BI, UK) and MAC using a non-elastic Lufkin® measuring tape. Measurements were taken three times, and mean values recorded following ISAK protocols. Mid-Arm Muscle Circumference (MAMC) and Mid-Arm Muscle Area (MAMA) were calculated using established equations [31], while corrected MAMA (cAMA) was computed following gender-specific adjustments [32]:


$$\begin{array}{c} MAMC=MAC-(\text{3}.\text{1}\text{4}\text{1}\text{5}\times TSF) \end{array}$$

$$\begin{array}{c} MAMA=\frac{{MAMC}^{\text{2}}}{\text{4}\times \text{3}.\text{1}\text{4}\text{1}\text{5}} \end{array}$$

$$\begin{array}{c} AMA\;(men)=\;MAMA\;-\;\text{1}\text{0} \end{array}$$

$$\begin{array}{c} AMA\;(women)=\;MAMA\;-\;\text{6}.\text{5} \end{array}$$

### Clinical, biochemical, and dietary assessments

Clinical assessments included evaluation of weight change, gastrointestinal symptoms, subcutaneous fat loss (orbital, triceps, biceps), muscle wasting (deltoid), and signs of edema. Biochemical parameters were obtained from patient records; missing laboratory values were collected with assistance from dialysis staff and analyzed at accredited diagnostic centers. Three 24-hour dietary recalls were conducted for each patient: one dialysis day, one non-dialysis day, and one weekend day. Average nutrient intake was calculated to assess habitual dietary patterns. The collected variables were subsequently used to compute Malnutrition Inflammation Scores (MIS) and classify nutritional status. Nutrient intakes were analyzed using standard food composition tables and averaged across the three recall days to estimate usual dietary intake.

### Diagnosis of malnutrition

Nutritional status was assessed using both the International Society of Renal Nutrition and Metabolism (ISRNM) criteria and the Malnutrition Inflammation Score (MIS).

According to ISRNM recommendations [15] malnutrition (protein-energy wasting) was diagnosed when at least three of the following four domains were abnormal:

1. **Serum chemistry** (albumin or total cholesterol),
2. **Body mass** (BMI, unintentional weight loss, or fat percentage changes),
3. **Muscle mass** (MAMC decline or reduced creatinine appearance),
4. **Dietary intake** (unintentional low protein or calorie intake).

The MIS consists of 10 components, including weight change (end dialysis dry weight), dietary intake, gastrointestinal symptoms, functional capacity (nutritionally related), co-morbidity, subcutaneous fat loss, muscle wasting, BMI, serum albumin, and total iron-binding capacity (TIBC). Each component is scored from 0 (normal) to 3 (severe), with a total score ranging from 0 to 30. For MIS, scores <5 indicated normal nutritional status, while scores ≥5 denoted malnutrition. The MIS comprises 10 components, each graded 0–3, producing a total score from 0 (normal) to 30 (severe malnutrition) [33,34].

### Statistical analysis

The Data were analyzed using SPSS version 26. Cases with missing values for variables required in a specific analysis were excluded from that analysis. Descriptive statistics were used to summarize all variables. The Shapiro-Wilk test assessed normality. Due to the distribution of variables, non-parametric tests were applied where appropriate. Binary logistic regression was performed to identify predictors of malnutrition. Multicollinearity among predictor variables was assessed prior to model development. Statistical significance was set at *p* < 0.05 with a 95% confidence interval. Model fit was evaluated using the Hosmer-Lemeshow goodness-of-fit test [35].

### Ethical considerations

All participants provided written informed consent prior to data collection. Ethical approval was granted by the Research Cell Committee of Noakhali Science and Technology University (NSTU/RC-FTNS/MS/21/71). Permissions were also obtained from all participating dialysis centers in the four study districts.

## Results

### Baseline characteristics

The baseline clinical and nutritional characteristics of the study cohort are summarized in **Table 1**. The study included 181 maintenance hemodialysis patients, with a nearly balanced gender distribution (96 males, 85 females). The median age was 48 years (IQR 38–60), and patients had a median dialysis vintage of 34 months (IQR 16–54). Most anthropometric measures differed significantly by gender: females had lower height and weight but higher TSF and MAC values than males (p < 0.05). BMI, MAMC, and cAMA showed modest differences, while biochemical parameters-including hemoglobin, serum glucose, sodium, potassium, and albumin-were largely comparable between genders. Notably, serum phosphorus was higher in females (p = 0.013). These data provide a comprehensive baseline profile of the cohort’s clinical and nutritional status, highlighting gender-specific variations relevant for subsequent analyses.

**Table 1:** Baseline clinical and nutritional characteristics of the study cohort.

Parameters	Gender			*p-value*
	All Patients (181) (n (%)/IQR)	Male (96) (n (%)/IQR)	Female (85) (n (%)/IQR)	
**Demographics**				
Age	48 (38–60)	48 (37.3–60)	50 (38.5–57.5)	.983
Dialysis Duration (hours)	4 (3.5–4)	4 (3.8–4)	4 (3.5–4)	.462
Dialysis Vintage (months)	34 (16–53.8)	35 (18–59)	25 (13–44)	***.031***
HH Income	3(2–4)	3(2–4)	3(2–4)	.907
Dialyzer Reuse	1 (0–2)	1 (0–2)	1 (0–2)	.274
**Anthropometrics**				
Height (cm)	162 (154.9–168.2)	167.6 (165–170)	154.9 (152–157.5)	***.000***
Body Weight (kg)	58 (52–65)	60 (56–67)	55 (49–62)	***.000***
BMI (kg/m^2^)	22.1 (20.3–24.7)	21.9 (20–24.1)	22.9 (20.6–25.3)	***.039***
MAC (cm)	26 (23.6–28.5)	25.1 (22.9–27.6)	27.4 (24.3–29.4)	***.002***
TSF (mm)	14.1 (8.4–19.1)	9.5 (7.3–15)	17.5 (14.2–21.9)	***.000***
MAMC (cm)	21.6 (20.2–23.1)	21.7 (20.5–23.3)	21.5 (19.8–22.9)	.193
cAMA (cm^2^)	28.4 (23.4–34.5)	27.7 (22.9–33.8)	29.6(24.1–35.1)	.256
**Biochemical**				
Hemoglobin (g/dL)	8.9 (7.6–10.2)	8.5 (7.6–10.2)	9 (7.6–10.3)	.413
Sr. glucose (mg/dL)	130.8 (95.3–201.5)	136.5 (98.6–200.7)	123.2 (92.9–192.5)	.204
TIBC (mg/dL)	223.5(193.3–257.5)	223.5(195.6–269.7)	223 (193–254)	.671
Sr. Sodium (mmol/L)	136 (133–138.6)	136(133.8–139)	135(132.8–138)	.188
Sr. Potassium (mmol/L)	4.6 (4.01–5.4)	4.6 (4–5.2)	4.6 (4.1–5.4)	.750
Sr. Phosphorus (mg/dL)	5 (3.6–6.2)	4.3 (3.1–5.6)	5.6 (4.4–6.2)	***.013***
Sr. Albumin (g/dL)	3.6 (3.4–3.9)	3.6 (3.4–3.9)	3.6 (3.4–3.8)	.809
Sr.TC (mg/dL)	146.9 (119.9–174)	139.6 (112.1–174)	150.8(131.1–174)	.055

Data were obtained from 181 maintenance hemodialysis patients across four districts in Bangladesh (Dhaka, Mymensingh, Cumilla, and Noakhali). Continuous variables are presented as median (interquartile range), and categorical variables as frequency (percentage). Group comparisons were performed using the Mann-Whitney U test, nonparametric independent samples t-test, or one-way ANOVA, as appropriate. A p-value <0.05 was considered statistically significant. HH income = household income; BMI = body mass index; MAC = mid-arm circumference; TSF = triceps skinfold thickness; MAMC = mid-arm muscle circumference; cAMA = corrected arm muscle area; TIBC = total iron-binding capacity; Sr. = serum; TC = total cholesterol.

The prevalence of malnutrition among the study cohort is summarized in **Table 2**. According to ISRNM criteria, 58.0% of patients had low BMI (<23 kg/m^2^), 60.8% had hypoalbuminemia (<3.8 g/dL), and 60.2% exhibited reduced muscle mass based on mid-arm muscle circumference. Inadequate dietary intake was observed in 42.5% for energy (<25 kcal/kg/day) and 31.5% for protein (<0.8 g/kg/day). Using the MIS, 72.9% of patients were classified as malnourished (MIS ≥ 5). A moderate positive correlation was observed between ISRNM and MIS scores (r = 0.282, p < 0.001), indicating consistency between these two validated nutritional assessment methods. These findings highlight the high burden of malnutrition in this population and support the use of multiple assessment tools for comprehensive evaluation in clinical practice.

**Table 2:** Prevalence of malnutrition among hemodialysis patients based on ISRNM criteria and malnutrition inflammation score (MIS), and their correlation.

Methods for nutritional Assessment	Classification		Frequency (%)
**ISRNM**	1. Body mass	BMI < 23 (kg/m^2^)	105 (58.0)
	2. Serum Chemistry	Sr. albumin<3.8 (g/dL)	110 (60.8)
		Total Cholesterol <100 (mg/dL)	23(12.7)
	3. Muscle mass (Mid-arm-muscle-circumference)	Mid arm muscle circumference below 10% of the 50^th^ percentile of reference US population	109 (60.2)
	4. Dietary Intake	Dietary Energy Intake <25 (kcal/kg-BW/day)	77 (42.5)
		Dietary Protein Intake <0.8 (g/kg-BW/day)	57 (31.5)
**MIS Score**	Score more than or equal to 5 (MIS ≥ 5)		132 (72.9)
**Correlation between the ISRNM and MIS**			r = 0.282 *p*=<*0.001*^*a*^

Malnutrition was assessed using both ISRNM criteria and the Malnutrition Inflammation Score (MIS). Values are presented as frequency (%). ISRNM = International Society of Renal Nutrition and Metabolism; MIS = Malnutrition Inflammation Score; BMI = body mass index (kg/m^2^); Sr. albumin = serum albumin (g/dL); TC = total cholesterol (mg/dL); MAMC = mid-arm muscle circumference; DEI = dietary energy intake (kcal/kg body weight/day); DPI = dietary protein intake (g/kg body weight/day). Spearman correlation coefficient (r) was used to evaluate the relationship between ISRNM and MIS scores; p < 0.001 was considered statistically significant.

**Fig 1:**
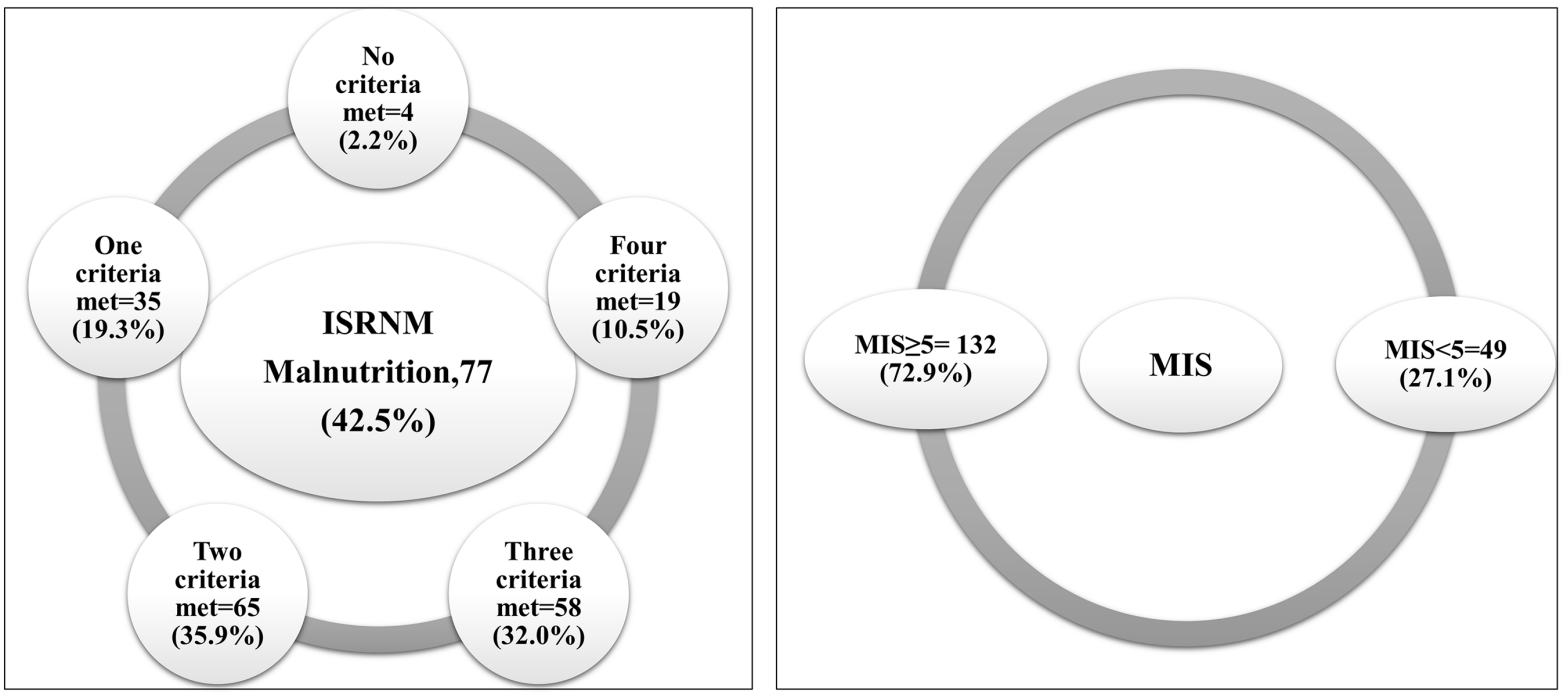
Prevalence of malnutrition among maintenance hemodialysis patients based on ISRNM criteria and malnutrition inflammation score (MIS). Data were collected from 181 maintenance hemodialysis patients across four districts in Bangladesh (Dhaka, Mymensingh, Cumilla, and Noakhali). Results are presented as frequency (%). ISRNM = International Society of Renal Nutrition & Metabolism; MIS = Malnutrition Inflammation Score.

Fig 1 illustrates the prevalence of malnutrition in the study cohort using both ISRNM criteria and MIS. According to ISRNM, only 2.2% of patients were well-nourished, while 10.5% exhibited severe malnutrition, meeting all four diagnostic criteria. Moderate risk (meeting two criteria) was observed in 35.9% of patients, high risk (three criteria) in 32.0%, and mild risk (one criterion) in 19.3%. Overall, 42.5% of patients were classified as malnourished based on ISRNM standards. Using MIS, 27.1% were categorized as having normal nutritional status, whereas 72.9% showed varying degrees of malnutrition, demonstrating concordance with ISRNM findings.

**Table 3** presents a detailed comparison between ISRNM classification and MIS in evaluating malnutrition among maintenance hemodialysis patients. ISRNM identified 58% of patients as malnourished based on body mass, serum chemistry, muscle mass, and dietary intake, while MIS (score ≥5) indicated malnutrition in 72.9% of patients. Age, dialysis vintage, dialyzer reuse, diabetes status, anthropometric measures (BMI, MAC, TSF, MAMC, cAMA), and serum albumin significantly differed between malnourished and non-malnourished groups across both methods. While both approaches were largely concordant, MIS detected a slightly higher prevalence, highlighting its sensitivity in capturing subclinical nutritional deficiencies. These findings underscore the importance of using complementary tools for accurate nutritional assessment in HD populations.

**Table 3:** Comparative analysis of nutritional status among hemodialysis patients using ISRNM classification and malnutrition inflammation score (MIS).

Parameters	ISRNM Classification			MIS Scale		
	Malnourished (n (%)/IQR)	Non-malnourished (n (%)/IQR)	P-value	MIS>=5 (n (%)/IQR)	MIS < 5 (n (%)/IQR)	P-value
**Demographics**						
**Age in years**						
18-35	20 (11.0)	15 (8.3)	.289	24 (13.5)	11 (6.2)	*.044*
36-55	31 (17.1)	54 (29.8)		57 (32.0)	26 (14.6)	
> 55	26 (14.4)	35 (19.3)		51 (28.7)	5 (5.1)	
Dialysis Duration (hours)	4 (3.5–4.0)	4 (3.8–4.0)	.553	4 (3.5–4.0)	4 (3.9–4.0)	.482
Dialysis Vintage (months)	34 (15–54.5)	34 (16–54)	.900	35 (17–59)	22.5 (10.6–39)	*.006*
**HH Income**						
Very Poor, < 5000 BDT	10 (5.5)	9 (5.0)	.084	17 (19.6)	2 (1.1)	.159
Poor, 5000 to 10,000 BDT	17 (9.4)	19 (10.5)		25 (14.0)	10 (5.6)	
Moderate, 10,000 to 20,000 BDT	29 (16.0)	34 (18.8)		48 (27.0)	15 (8.4)	
High, > 20,000 BDT	21 (11.6)	42 (23.2)		42 (23.6)	19 (10.7)	
**Dialyzer Reuse**						
≤ 2	63 (34.8)	99 (54.7)	*.004*	116 (65.2)	43 (24.2)	.292
> 2	14 (7.7)	5 (2.8)		16 (9.0)	3 (1.7)	
**Diabetes**						
Yes	18 (11.5)	35 (22.4)	*.029*	34 (21.9)	19 (12.3)	*.028*
No	54 (34.6)	49 (31.4)		82 (52.9)	20 (12.9)	
**Anthropometrics**						
Height (cm)	165 (160–170)	160 (154–166.1)	*.000*	162 (154.9–167.3)	162.6(154.9–170)	.625
Post dialysis Body Weight (kg)	56.1 (48.6–60.5)	60 (54–68.5)	*.000*	57 (51–63)	62.5(56.8–72.3)	*.000*
BMI (kg/m^2^)	20.6 (18.9–21.9)	24 (21.6–26.2)	*.000*	21.9 (19.5–24.2)	23.3 (21.3–27.2)	*.000*
**WHO criteria-based BMI classification (Kg/m**^**2**^)						
Underweight	17 (9.6)	7 (3.9)	*.000*	24 (13.7)	0 (0)	
Normal	56 (31.5)	57 (32.0)		81 (46.3)	29 (16.6)	
Overweight	4 (2.2)	27 (15.2)		20 (11.4)	11 (6.3)	
Obese	0 (0)	10 (5.6)		4 (2.3)	6 (3.4)	
MAC (cm)	23.6(22.1–26)	28.3(25.6–30.2)	*.000*	25.3(22.8–27.9)	28.4 (25.6–30.2)	*.000*
TSF (mm)	9 (6.9–14.8)	17.5 (12.4–21.3)	*.000*	12.6 (8–17.4)	18 (10.3–20.8)	*.004*
MAMC (cm)	20.5(19.1–21.8)	22.6 (21.1–23.8)	*.000*	21.1 (19.7–22.9)	22.7 (21.6–24.3)	*.000*
cAMA (cm^2^)	24.2 (20–29.)	32.5 (26.6–36.3)	*.000*	27.5 (22.9–33.3)	33.4 (26.11–37.9)	*.004*
**Biochemical**						
Hemoglobin (g/dL)	8.9 (7.6–10.3)	8.6 (7.6–10.1)	.543	8.8 (7.6–9.9)	9.4 (7.8–10.8)	.061
Sr. glucose (mg/dL)	125.5 (92.1–161)	137.5 (97.8–203.5)	.092	127.5 (93.2–194.5)	149.3 (103.4–205.3)	.054
TIBC (mg/dL)	228.6 (189.3–257.2)	219.7 (198.1–260.3)	.977	220.9(188–251.5)	247.6 (205.7–272.4)	*.016*
Sr. Sodium (mmol/L)	135.2 (132.4–138.6)	136(134–138.9)	.175	136(132.8–138.7)	137(134–138.7)	.287
Sr. Potassium (mmol/L)	4.6 (3.9–5.3)	4.6 (4.1–5.4)	.422	4.6(4–5.3)	4.8(4.0–5.7)	.348
Sr. Phosphorus (mg/dL)	5.1 (2.9–6.2)	4.9 (3.8–6.1)	.700	4.8 (3.3–5.9)	5.3(4.6–6.5)	.085
Sr Albumin (g/dL)	3.5(3.1–3.6)	3.8 (3.5–4)	*.000*	3.5(3.2–3.8)	3.8(3.7–4.1)	*.000*
Sr.TC (mg/dL)	144.5 (110.5–170.1)	154.7 (123.7–175.9)	*.038*	146 (116–174)	162.7 (128.6–175.4)	.193

Results are presented as frequency (%) or median (interquartile range). Nonparametric Mann-Whitney U test and one-way ANOVA were used to compare groups. P < 0.05 was considered statistically significant. Abbreviations: MIS = Malnutrition Inflammation Score; HH income = Household income; BMI = Body Mass Index; MAC = Mid-arm circumference; TSF = Triceps skinfold thickness; MAMC = Mid-arm muscle circumference; cAMA = Corrected arm muscle area; Hb = Hemoglobin; Sr. = Serum; TIBC = Total iron-binding capacity; TC = Total cholesterol.

**Table 4** presents a comprehensive analysis of dietary intake, including macronutrients and minerals, in relation to the nutritional status of maintenance hemodialysis patients. Nutrient intake was assessed using 3 days 24-hour dietary recall, with results expressed as mean ± SD. Among the 181 patients initially enrolled, 23 were excluded due to misreporting, including 3 cases of overreporting and 20 cases of underreporting. Consequently, a total of 158 patients (84 males and 74 females) were identified as acceptable reporters based on the Goldberg index, using a cutoff of <0.8 for underreporting and >1.3 for overreporting [36,37].

**Table 4:** Dietary macro and micronutrient intake among hemodialysis patients based on nutritional status assessed by ISRNM and MIS.

Parameters	ISRNM Classification				Malnutrition based on MIS scale			
	Malnourished (66)	Non-malnourished (92)	P-value	%MD^a^	MIS>=5 (112)	MIS < 5 (43)	P-value	%MD^b^
**Macronutrients**								
Energy (kcal)	1449.4 ± 260.6	1646.9 ± 346.8	<0.001	13.6	1485.1 ± 266.4	1775.8 ± 377.7	<0.001	19.6
Protein (g)	50.5 ± 13.0	67.8 ± 22.5	<0.001	34.3	53.3 ± 12.4	79.6 ± 26.5	<0.001	49.3
DEI (Kg/BW/Day)	26.7 ± 5.8	27.1 ± 5.5	*0.632*	1.5	26.6 ± 6.0	27.8 ± 4.2	*.244*	4.5
DPI (Kg/BW/Day)	0.9 ± 0.3	1.1 ± 0.3	<0.001	22.2	0.9 ± 0.3	1.2 ± 0.3	<0.001	33.3
Carbohydrate (g)	208.6 ± 46.1	223.7 ± 51.7	0.060	7.2	210.0 ± 45.2	235.9 ± 55.8	0.003	12.3
Fat (g)	42.5 ± 13.8	49.4 ± 15.5	0.005	16.2	44.4 ± 13.1	52.7 ± 18.6	0.002	18.7
Total Fiber (g)	16.4 ± 5.4	18.3 ± 4.9	0.028	11.6	16.9 ± 5.4	19.1 ± 4.1	0.016	13.0
**Micronutrients (Minerals)**								
Calcium (mg)	372.3 ± 153.9	468.2 ± 259.7	.043	25.8	388.3 ± 160.4	517.7 ± 278.6	0.013	33.3
Iron (mg)	19.6 ± 13.4	19.7 ± 12.9	0.980	0.5	19.1 ± 12.9	21.8 ± 15.2	0.567	14.1
Phosphorus (mg)	772.2 ± 172.7	940.5 ± 271.9	<0.001	21.8	797.5 ± 166.3	1053.4 ± 320.8	<0.001	31.1
Potassium (mg)	1493.6 ± 371.1	1836.5 ± 493.8	<0.001	22.9	1558.8 ± 371.1	2041.0 ± 522.2	<0.001	30.9
Sodium (mg)	1895.1 ± 818.0	2142.4 ± 654.9	0.037	13.0	1974.0 ± 750.7	2237.7 ± 675.6	0.046	13.3
Zinc (mg)	6.9 ± 2.1	8.5 ± 3.1	<0.001	23.2	7.2 ± 2.0	9.6 ± 3.8	<0.001	33.3
Magnesium (mg)	241.7 ± 53.9	286.9 ± 78.1	<0.001	18.7	249.5 ± 66.4	316.1 ± 65.8	<0.001	26.7

Results were presented as mean±SD. Significant differences of dietary intakes between groups were observed using Kruskal-Wallis one-way ANOVA with a p-value<0.05. Percentage mean difference (%MD) was individually computed using the formula: % MD^a^= (No malnutrition – malnutrition)/ malnutrition ×100 and % MD^b^=(MIS < 5-MIS ≥ 5)/MIS ≥ 5 × 100.

Dietary intake analyses demonstrated that malnourished patients consistently consumed less energy and protein than their well-nourished counterparts according to both ISRNM and MIS classifications. Based on ISRNM classification, malnourished patients exhibited significantly lower dietary energy intake (1449.4 ± 260.6 vs. 1646.9 ± 346.8 kcal, p < 0.001), representing a 13.6% lower energy intake. Similarly, dietary protein was 34.3% lower in malnourished patients (50.5 ± 13.0 vs. 67.8 ± 22.5 g/day, p < 0.001). Comparable findings were observed using MIS classification, where patients with MIS ≥ 5 reported 19.6% lower energy intake (1485.1 ± 266.4 vs. 1775.8 ± 377.7 kcal/day) and 49.3% lower protein intake (53.3 ± 12.4 vs. 79.6 ± 26.5 g/day) than those with MIS < 5 (both p < 0.001). Although dietary protein intake normalized to body weight remained significantly lower among malnourished participants under both classifications, dietary energy intake per kilogram body weight did not differ significantly. In addition to energy and protein, malnourished patients generally reported lower intakes of other macronutrients. Fat and dietary fiber intake were significantly lower under both ISRNM and MIS classifications, whereas carbohydrate intake differed significantly only under MIS classification (p = 0.003).

Among micronutrients, phosphorus, potassium, zinc, and magnesium intakes were 21.8%, 22.9%, 23.2%, and 18.7% lower among malnourished patients (all p < 0.001) under ISRNM classification, respectively. Similar but larger differences were observed using MIS, with reductions of 31.1%, 30.9%, 33.3%, and 26.7%, respectively. Calcium intake was also significantly lower in malnourished patients according to both classifications, whereas iron intake showed no significant association with nutritional status.

Building upon the demographic, anthropometric, biochemical, and dietary analyses presented in Tables 1-4 and Fig 1, **Table 5** highlights the independent predictors of malnutrition among maintenance hemodialysis patients, where malnutrition was defined based on ISRNM criteria. Variables with p < 0.25 in bivariate analyses-age, dialyzer reuse, diabetes mellitus, and BMI-were included in the multivariate logistic regression.

**Table 5:** Multivariable logistic regression of factors associated with malnutrition in maintenance hemodialysis patients.

Variable	Malnutrition		COR (95%CI)	AOR (95%CI)	*p- Value* for AOR
	Yes, N (%)	No, N (%)			
**Age**					
18-35	20 (11)	15 (8.3)	I*		
36-55	31 (17.1)	54 (29.8)	0.4 (0.2, 0.9)	0.4 (0.2, 1.0)	.07
> 55	26 (14.4)	35 (19.3)	0.5 (0.2, 1.3)	0.9 (0.3, 2.7)	.96
**Family Monthly income**					
Very Poor, < 5000 BDT	10 (5.5)	9 (5)	I*		
Poor, 5000 to 10,000 BDT	17 (9.4)	19 (10.5)	0.8 (0.2, 2.5)		
Moderate, 10,000 to 20,000 BDT	29 (16)	34 (18.8)	0.8 (0.3, 2.1)		
High, > 20,000 BDT	21 (11.6)	42 (23.2)	0.5 (0.2, 1.3)		
**Dialysis frequency**					
1/weeks	1 (0.6)	2 (1.1)	I**		
2/weeks	71 (39.2)	74 (40.9)	1.9 (0.2, 21.4)		
3/weeks	5 (2.8)	28 (15.5)	0.4 (0.0, 4.7)		
**Dialysis Vintage**					
3 months to 30	37 (20.6)	48 (26.7)	I		
31 to 58	23 (12.8)	33 (18.3)	0.9 (0.5, 1.8)		
59 to 86	14 (7.8)	17 (9.4)	1.1 (0.5, 2.4)		
> 86	3 (1.7)	5 (2.8)	0.8 (0.2, 3.5)		
**Dialyzer Reuse**					
≤ 2	63 (34.8)	99 (54.7)	I**		
> 2	14 (7.7)	5 (2.8)	4.4 (1.5, 12.8)	5.8 (1.2, 28.7)	*.02*
**Co-morbidity (Diabetes mellitus)**					
No	18 (11.5)	35 (22.4)	I**		
Yes	54 (34.6)	49 (31.4)	0.5 (0.2, 0.9)	0.5 (0.2, 1.0)	.13
**BMI**					
< 18.5	17 (9.6)	7 (3.9)	I**		
18.5 to 24.9	56 (31.5)	57 (32)	0.4 (0.2, 1.1)	0.2 (0.1, 0.8)	*.02*
25 to 30	4 (2.2)	27 (15.2)	0.1 (0.0, 0.2)	0.0 (0.0, 0.2)	*<0.001*
> 30	0 (0)	10 (5.6)	0.0 (0,0)	0.0 (0, 0)	.99

COR: Crude Odds Ratio; AOR: Adjusted Odds Ratio; BMI: Body Mass Index. Notes: *p < 0.25, *p ≤ 0.05, reference groups indicated by I. Model fit: Hosmer-Lemeshow chi-square = 3.1, p = 0.8; omnibus test p < 0.001.

The results demonstrate that patients who reused the dialyzer more than twice had a 5.8-fold higher likelihood of malnutrition (AOR = 5.8; 95% CI: 1.2–28.7; p = 0.02) compared to those reusing it ≤ 2 times. Patients with moderate BMI (18.5–24.9 kg/m^2^) were 0.2 times less likely to have malnutrition than underweight patients (AOR = 0.2; 95% CI: 0.1–0.8; p < 0.001). Age and diabetes mellitus were also included as predictors, although age > 55 years did not reach statistical significance (AOR = 0.9; p > 0.05). These findings underscore the multifactorial etiology of malnutrition in dialysis patients, emphasizing both treatment-related and patient-specific factors as key considerations for nutritional intervention strategies.

## Discussion

In this study, we evaluated the nutritional status of HD patients across four districts of Bangladesh using both ISRNM criteria and MIS. Gender-specific differences were observed in height, body weight, BMI, MAC, and TSF, all significantly associated with malnutrition, consistent with prior studies highlighting body composition variations between male and female dialysis patients [38]. Female patients demonstrated higher serum total cholesterol and phosphorus levels, correlating with malnutrition, while other biochemical markers showed no significant gender differences, aligning with findings from Jordan [39].

The prevalence of malnutrition in our cohort was substantial, with 42.5% of patients meeting ISRNM criteria, comparable to the 40% reported in Spain [16]. Only 2.2% of patients were exceptionally well-nourished, meeting none of the criteria, while more than 40% were classified as malnourished based on SGA assessment [40]. According to ISRNM criteria, 10.5% had severe malnutrition (meeting all four criteria), 32.0% were at high risk (three criteria), and 35.9% had moderate risk (two criteria), aligning with findings from [21], who reported that over 70% of HD patients exhibited malnutrition or PEW, albeit with a higher proportion of normal nutritional status than in our study. Global estimates suggest that 28–54% of dialysis patients experience PEW [41]. In terms of BMI, 58% of patients were classified as malnourished (<23 kg/m^2^) per ISRNM criteria. Interestingly, a Brazilian study reported that HD patients with BMI < 23 kg/m^2^ showed no energy depletion, whereas those with BMI > 23 kg/m^2^ had more inflammation, likely due to increased adiposity [42] This highlights the need for population-specific BMI thresholds, particularly in Asian cohorts [43].

Serum chemistry indicators revealed that 60.8% had albumin <3.8 g/dL and 12.7% had total cholesterol <100 mg/dL; however, serum albumin may reflect inflammation more than nutritional status [22]. Dietary assessment showed that 47% had DEI < 25 kcal/kg-BW/day and 20.4% had DPI < 0.8 g/kg-BW/day. Additionally, 72.9% were classified as malnourished based on MIS (≥5), and 60.2% showed low mid-arm muscle circumference. These results are consistent with previous Bangladeshi findings [34], and other international reports, with malnutrition prevalence ranging 20–75% in HD populations [15,44,45]. ISRNM and MIS scores were positively correlated (r = 0.282, p = 0.000), demonstrating consistency between assessment tools. Similarly, SGA and Malnutrition Universal Screening Tool (MUST) [46].

The higher prevalence of malnutrition identified by MIS (72.9%) compared to ISRNM (42.5%) likely reflects methodological differences between the two assessment tools. MIS incorporates both objective biochemical parameters and subjective clinical components, including dietary intake, gastrointestinal symptoms, functional capacity, and comorbidity burden, as well as markers related to inflammation. In contrast, ISRNM criteria rely primarily on objective indicators across defined domains such as body mass, muscle mass, serum chemistry, and dietary intake. As MIS captures broader clinical and inflammatory aspects of PEW, it may be more sensitive in detecting early or subclinical malnutrition, particularly in populations with a high inflammatory burden.

Age-related differences indicated that older adults (>55 years) tended to have higher MIS scores, although differences were not statistically significant, corroborating previous evidence that aging and comorbidities increase malnutrition risk [47,48]. Dialyzer reuse >2 was significantly associated with higher malnutrition prevalence (p < 0.05). In Bangladesh, dialyzer reuse is often influenced by economic constraints and institutional practices rather than individual clinical or nutritional conditions. Many patients reuse dialyzers to reduce the financial burden of long-term dialysis treatment, as out-of-pocket healthcare expenses remain substantial in resource-limited settings. Therefore, dialyzer reuse can also reflect underlying socioeconomic factors that could contribute to inadequate dietary intake or limited access to supportive healthcare services. This can partly explain the observed association between dialyzer reuse and malnutrition. Further studies are needed to clarify the causal mechanisms underlying this relationship. On the other hand, association between dialyzer reuse and malnutrition can be biologically plausible [49]. Repeated use of dialyzers can reduce membrane efficiency, leading to inadequate toxin clearance [50], while also increasing exposure to bioincompatible materials that can trigger chronic inflammation [51]. Additionally, reuse practices can increase infection risk [52] and are often driven by economic constraints, which may also be linked to poorer overall nutritional status and healthcare access.

We observed that hemodialysis patients, malnutrition defined by both ISRNM and MIS criteria was highly prevalent and strongly associated with lower dietary intakes of energy, protein, fat, fiber, and several key micronutrients. Malnourished patients consistently consumed 13–20% less total energy and approximately one‑third to one‑half less protein than their well‑nourished counterparts. A recent study in Dhaka assessed protein–energy wasting (PEW) in HD patients, found that most patients, regardless of PEW status, failed to meet recommended intakes for energy, protein, fiber and several micronutrients, sometimes reaching only 10–40% of guideline targets [34]. Another study in Vietnam reported an average energy intake of 21.5 kcal/kg/day, with only 3.9% meeting recommended levels; average protein intake was 1.0 g/kg/day, with only about 10.5% meeting recommendations [53]. Furthermore, malnutrition in HD patients is multifactorial and may occur despite apparently adequate intake due to inflammation, metabolic disturbances, hormonal imbalance, and dialysis-related nutrient losses [54]. Beyond energy and protein, this study found that malnourished patients had significantly lower intakes of fat and dietary fiber under both classifications, indicating an overall reduction in diet quantity and quality. The most striking micronutrient findings were 20–30% reductions in phosphorus, potassium, zinc, magnesium, and calcium intake among malnourished individuals. These reductions likely mirror the lower consumption of protein‑rich foods and may indicate overall poor intake of nutrient‑dense foods such as dairy products, meat, legumes, and certain vegetables [53]. However, dialysis patients are commonly advised to restrict intake of certain minerals, particularly sodium and phosphorus, to limit volume overload and hyperphosphatemia, because impaired renal excretion and incomplete removal by dialysis predispose to fluid retention and mineral imbalance [55].

Multivariate analysis identified key independent predictors of malnutrition: dialyzer reuse >2, BMI, and diabetes mellitus status. Previous studies showed that diabetic hemodialysis patients have a higher incidence of protein malnutrition and poorer survival than non-diabetic patients [56]. However, a recent review on HD patients found minimal or multiple dialyzer reuse had no impact on body weight or serum albumin, which means reuse is not consistently linked to malnutrition [57]. These results underscore the multifactorial etiology of malnutrition among dialysis patients, integrating demographic, clinical, and dietary factors.

### Strengths and limitations

This study offers several strengths including the simultaneous use of two established nutritional assessment methods (ISRNM and MIS), and the evaluation of both macronutrient and micronutrient intake, which remains understudied in Bangladesh. Although ISRNM and MIS are internationally established tools, their formal validation in Bangladeshi populations remains limited. Furthermore, the identification of modifiable risk factors, such as dialyzer reuse and inadequate dietary intake, provides clinically relevant targets for intervention and highlights potential opportunities to improve nutritional care among HD patients.

Despite these strengths, several limitations are considered when interpreting the findings. The cross-sectional design also prevents assessment of temporal relationships between potential risk factors and malnutrition, as exposure and outcome variables were assessed at a single point in time. The use of convenience sampling may have introduced selection bias, as patients who agreed to participate or attended the selected dialysis facilities may differ systematically from the broader hemodialysis population. Consequently, the findings should be generalized with caution to all hemodialysis patients in Bangladesh or other resource-limited settings. In some facilities, patient records were not consistently available; therefore, certain clinical and dietary variables were collected directly from patients during dialysis sessions, which may introduce recall or reporting bias. Future studies using larger, multicenter samples and probabilistic sampling techniques are recommended to improve representativeness and strengthen the robustness of findings. Despite these limitations, the study provides novel, comprehensive insights into the nutritional status of HD patients in Bangladesh, establishing a foundation for future longitudinal research and targeted interventions aimed at reducing morbidity and mortality in this high-risk population.

## Conclusions

This comprehensive study, employing both ISRNM criteria and the Malnutrition Inflammation Score (MIS), reveals a strikingly high prevalence of malnutrition-42.5%-among hemodialysis patients in Bangladesh. By integrating anthropometric, biochemical, and dietary assessments, the research highlights the multifaceted nature of malnutrition in this high-risk population. Key indicators-serum albumin, BMI, and MIS scores-emerge as practical, cost-effective tools for early detection and timely intervention. Demographic and clinical factors-including age, gender, dialyzer reuse, and micronutrient intake-significantly influence nutritional status, with older adults and patients with lower BMI being particularly vulnerable. Importantly, combining multiple, correlated assessment methods provides a robust framework for identifying at-risk patients and guiding personalized nutritional interventions. These findings underscore an urgent need for targeted clinical nutrition strategies, especially in resource-limited settings, to reduce morbidity, enhance quality of life, and improve survival among dialysis populations. They also offer a strong rationale for future research aimed at optimizing malnutrition assessment and intervention in similar high-risk cohorts worldwide.

## Supporting information

S1 File Data. (XLSX)
