# Supplementary material for: Malnutrition in maintenance hemodialysis: Prevalence, patterns, and clinical implications in Bangladesh
Source: PLoS One. 2026 Jul 17;21(7):e0354173. doi: 10.1371/journal.pone.0354173 (PMC13379082; doi:10.1371/journal.pone.0354173)
Supplement: Attachment — Submitted filename: Response to Reviewers.docx [file pone.0354173.s003.docx]

April 21, 2026

Editor-in-Chief,

PLOS ONE journal.

We would like to thank you and the reviewers for the careful evaluation of our manuscript and for providing constructive and insightful comments. We have carefully revised the manuscript in response to all reviewer suggestions and believe that these revisions have significantly improved the clarity, methodological transparency, and scientific quality of the paper.

In particular, we have clarified the definition and interpretation of malnutrition using both ISRNM and MIS criteria, expanded the discussion regarding the observed differences between these assessment tools, and provided further explanation regarding the potential mechanisms linking dialyzer reuse with malnutrition, including considerations related to dialysis adequacy, inflammation, infection risk, and socioeconomic factors in resource-limited settings. We have also improved the interpretation of dietary intake findings, acknowledging the limitations of short-term dietary recall and the multifactorial nature of protein–energy wasting in hemodialysis patients.

Furthermore, we have strengthened the Strengths and Limitations section by addressing the implications of the cross-sectional design, convenience sampling, and data collection challenges in dialysis centers. Minor revisions were also made to ensure consistency in terminology and units throughout the manuscript.

We believe that the revised manuscript provides important and novel evidence on the burden and associated factors of malnutrition among maintenance hemodialysis patients in Bangladesh, particularly through the simultaneous application of ISRNM and MIS criteria and the evaluation of macro- and micronutrient intake, which remains understudied in this population. These findings may contribute to improved nutritional assessment and clinical management strategies for dialysis patients in resource-limited settings.

We sincerely appreciate the opportunity to revise our manuscript and hope that the revised version meets the journal’s standards for publication.

Thank you for your consideration.

Sincerely,

On behalf of all author

Shakil Ahmed

**Response to Reviewer 1:**

| **Comments from Reviewer 1:** | **Response to Reviewer:** |
| --- | --- |
| 1. The authors showed the clinical characteristics of malnutrition with maintenance HD patients in Bangladesh. This paper is original since no data has yet been presented. I have some comments and concerns regarding this manuscript. | We sincerely thank the reviewer for the insightful and constructive comments. We have carefully revised the manuscript to address all concerns. Our detailed responses are provided below. |
| 2. First of all, the number of the study patients was relatively small. In addition, the study design was cross-sectional study. They were a critical limitation of this study. | Thank you for this insightful comment. We acknowledge that the relatively small sample size is a limitation of this study. The smaller number of participants was mainly due to practical challenges, including limited willingness of patients to participate in research surveys, restricted access to dialysis centers, incomplete medical record documentation, and the need for offline, face-to-face data collection during dialysis sessions. We have now clarified this point in the “Strengths and Limitations” section. In addition, we have cited comparable state-of-the-art studies [25, 33, 45, 48] with similar or less sample sizes.  Regarding study design, the aim of this study was to assess the prevalence of malnutrition among hemodialysis patients in a resource-limited setting in Bangladesh. Therefore, a cross-sectional design is appropriate for generating primary data and estimating disease prevalence, as it provides a snapshot of a specific population at a single point in time in a relatively fast and cost-effective manner. |
| 3. The definition of malnutrition is important in this study. However, I concerned that there is a possibility that its definition is not appropriate in this study. Table 4 shows that the daily energy intake (DEI) and the daily protein intake (DPI) were significantly higher in patients with malnutrition compared to those without malnutrition. | We appreciate this important observation and thank you for your comment. In this study, malnutrition was defined using established ISRNM criteria and the MIS, which incorporate anthropometric, biochemical, clinical, and dietary indicators rather than relying solely on dietary intake. Therefore, we have revised the Results and Discussion sections to clarify the interpretation of Table 4.  Additionally, we would like to acknowledge that we initially applied an incorrect cut-off point for under-reporting Goldberg Index = (Energy Intake / Basal Metabolic Rate) using >0.9. However, for hemodialysis (HD) patients, a study used cut-off is 0.76 [35]. We have now corrected this and recalculated the data accordingly, and the revised results have been updated in the manuscript. |
| Comments 3. The definition of malnutrition was not mentioned in Table 5. Did the authors conduct the validation of the ISRNM and MIS in Bangladesh patients? | We have now explicitly stated in Table 5 and the Methods section that malnutrition was defined based on ISRNM criteria. Regarding validation, both ISRNM and MIS are internationally established tools widely used in HD populations. However, we acknowledge that formal validation in Bangladeshi populations is limited and have added this as a limitation in the revised manuscript. |
| 4. The authors demonstrated that dialyzer reuse was significantly correlated with malnutrition. I concerned that there is a possibility that the bias may be present. How do the physicians decide to reuse the dialyzer in Bangladesh? | Thank you for this important observation. In Bangladesh, dialyzer reuse is primarily influenced by economic constraints and institutional practices rather than patient nutritional status. Patients often reuse dialyzers to reduce the financial burden of repeated dialysis sessions. Therefore, dialyzer reuse may also reflect underlying socioeconomic conditions that are associated with increased risk of malnutrition. We have clarified this point in the Discussion section and acknowledged the possibility of residual confounding. |
| 5. In the introduction, the authors mentioned that the prevalence of malnutrition is 42.7% in the world by referring only one study. The universal data should be mentioned on the basis of the several references. | Thank you for this valuable suggestion. We have revised the Introduction to reflect variability in global prevalence of malnutrition among hemodialysis patients by citing multiple references rather than relying on a single estimate. |
| 6. The description of MIS components should be mentioned in the manuscript. | Thank you for your insightful comment. We have expanded the Methods section to clearly describe the 10 components of the MIS scoring system. |
| 7. The results of creatinine and eGFR should be removed in Table because all the study patients undertook maintenance hemodialysis. | Thank you for this valuable comment. We agree that creatinine and eGFR are not clinically informative parameters in maintenance hemodialysis patients. Therefore, we have removed these variables from Table 1 and Table 3 and revised the corresponding text accordingly. |

**Response to Reviewer 2:**

| **Comments from Reviewer 2:** | **Response to Reviewer 2:** |
| --- | --- |
| 1. This paper comprehensively examines the critical and unresolved issue of malnutrition among HD patients in Bangladesh using reliable assessment tools, demonstrating high academic and clinical value. By focusing on the key points mentioned above for revision, it is expected to achieve a level of completeness sufficient for publication in an international journal. | We thank the reviewer for the valuable suggestions, which have significantly improved the manuscript. |
| 2. Study design and sampling bias  The use of convenience sampling limits representativeness and external validity.  You may discuss the impact on generalizability and propose probabilistic sampling in future studies. | Thank you for this valuable comment. We have now clarified in the Strengths and Limitations section that convenience sampling and recruitment from a limited number of dialysis centers may affect representativeness of the findings. We also highlighted that future studies using larger, multicenter samples and probabilistic sampling approaches are recommended to improve robustness and representativeness. |
| 3. Limited discussion on discrepancy between ISRNM and MIS  The large difference in malnutrition prevalence between ISRNM and MIS warrants deeper methodological and clinical discussion.  Please clarify differences in sensitivity, subjectivity, and inflammatory components of MIS. | Thank you for this important suggestion. We have expanded the Discussion section to clarify the discrepancy in malnutrition prevalence between ISRNM and MIS. MIS incorporates both objective biochemical parameters and subjective clinical components, including factors related to inflammation, functional status, and comorbidity burden, which can increase its sensitivity in detecting early or subclinical protein-energy wasting. In contrast, ISRNM relies primarily on objective criteria across defined nutritional domains. These methodological differences are explained the higher prevalence of malnutrition identified by MIS in our study. |
| 4. Effect of dialyzer reuse as a predictor  While statistically significant, the biological plausibility is insufficiently explored.  Please discuss the mechanisms such as inflammation, toxin clearance, and infection risk. | Thank you for this valuable comment. We have expanded the Discussion section to further explore the biological plausibility of the association between dialyzer reuse and malnutrition. Repeated use of dialyzers can reduce membrane efficiency, potentially leading to inadequate toxin clearance, while exposure to bioincompatible materials can contribute to chronic inflammation. In addition, reuse practices can increase infection risk. These clinical factors, together with underlying economic constraints influencing reuse practices, can contribute to increased risk of protein-energy wasting among hemodialysis patients. The relevant discussion has been added to improve interpretation of this finding. |
| 5. Minor Revision  Please recheck some terminology and units' error  Ex.Inconsistent use of “Sr.” vs “Serum” or " μmol//l". | Thank you for pointing this out. We have carefully reviewed the manuscript and corrected inconsistencies in terminology and unit formatting throughout the text, tables, and figures. In particular, abbreviations such as “Sr.” were replaced with the standardized term “Serum,” and unit formatting (e.g., μmol/L, mg/dL, kg/m²) was revised for consistency according to journal style. In the table we used Sr. shortform and its full form is mentioned in every table footnote. |
