# Supplementary material for: Malnutrition in maintenance hemodialysis: Prevalence, patterns, and clinical implications in Bangladesh
Source: PLoS One. 2026 Jul 17;21(7):e0354173. doi: 10.1371/journal.pone.0354173 (PMC13379082; doi:10.1371/journal.pone.0354173)
Supplement: Attachment — Submitted filename: Response_to_Reviewers_auresp_2.docx [file pone.0354173.s004.docx]

**Response to the Academic Editor**

*PLOS ONE*

**Manuscript:** Malnutrition in maintenance hemodialysis: Prevalence, patterns, and clinical implications in Bangladesh

June 21, 2026

Dear **Editor-in-Chief**,

We thank you for your continued assessment of our manuscript and for the opportunity to submit a revision. We have carefully considered the editorial decision and have focused this revision on strengthening the methodological interpretation and discussion, as advised. We note that the reviewers raised no further comments in this round; accordingly, we have addressed each of the Academic Editor's points in full below, and we provide a clear point-by-point response as requested. All changes are shown as tracked changes in the revised manuscript. Your comments are reproduced in italics (or quotation marks), followed by our responses and the location of each revision.

**1. Interpretation of dietary intake findings**

*“Although revised, the apparent inconsistency between higher intake and malnutrition status should be explained more clearly and cautiously.”*

**Response:** We are grateful for this observation. On re-examining our data we found that the earlier wording was inaccurate: malnourished patients did not have higher intake. As shown in Table 4, malnourished patients had significantly lower absolute energy and protein intakes than non-malnourished patients. However, energy intake expressed per kilogram of body weight (DEI) did not differ significantly between the groups (approximately 26–27 kcal/kg/day) and approached recommended levels. We have rewritten the relevant paragraph in the Discussion to state this accurately and cautiously, and to explain that malnutrition in hemodialysis is multifactorial and may occur despite seemingly adequate weight-adjusted intake owing to inflammation, metabolic and hormonal disturbances, and dialysis-related nutrient losses. We also clarify that nutritional status was interpreted using combined anthropometric, biochemical, and clinical indicators rather than dietary intake alone.

**2. Dialyzer reuse and confounding**

*“While socioeconomic context is mentioned, the potential residual confounding and causal interpretation should be further toned down.”*

**Response:** We have toned down the causal interpretation in the Discussion. The text now states explicitly that, because dialyzer reuse is closely tied to socioeconomic status in this setting, the observed association is likely subject to residual confounding and that a causal relationship cannot be inferred from these cross-sectional data. We have added that the estimate is statistically imprecise: only 19 patients reused dialyzers more than twice, producing a wide confidence interval (AOR = 5.8; 95% CI: 1.2–28.7). The finding is now described as hypothesis-generating, and we note that prospective studies measuring and adjusting for socioeconomic and inflammatory factors are needed. In addition, the proposed biological mechanisms (reduced membrane efficiency, exposure to bio-incompatible materials, inflammation, and infection risk) are now explicitly framed as hypothesized pathways that were not measured in the present study.

**3. Methodological limitations**

*“The implications of convenience sampling and cross-sectional design on generalizability should be emphasized more explicitly.”*

**Response:** We have expanded the Strengths and Limitations section. We now state that, because convenience sampling was used, the cohort is likely to over-represent more stable and treatment-adherent patients attending established urban dialysis centers, while under-representing those in primary or rural facilities, those not currently receiving dialysis, and those who declined participation; the direction and magnitude of this selection bias cannot be quantified. We state explicitly that the prevalence estimates and associations reported here should be interpreted as specific to the studied centers and should not be generalized to the national maintenance hemodialysis population. We further note that, combined with the cross-sectional design, these features mean the results describe associations at a single time point rather than causal or temporal relationships.

**4. Clarity and consistency**

*“Minor issues in terminology, unit presentation, and clarity of tables should be carefully rechecked.”*

**Response:** We have rechecked terminology, units, and tables throughout, and made the following corrections (all shown as tracked changes):

- Corrected a statement that “ISRNM identified 58% of patients as malnourished” (before Table 3); 58% was the proportion with low BMI (<23 kg/m²). The ISRNM malnutrition prevalence is 42.5% (meeting at least three of the four domains), and the text now reads accordingly.
- Reconciled the dietary-inadequacy percentages in the Discussion (previously stated as 47% and 20.4%) with Table 2, which reports 42.5% for energy intake <25 kcal/kg/day and 31.5% for protein intake <0.8 g/kg/day.
- Clarified that the figure of “more than 40% malnourished by SGA” refers to the cited Jordanian study, not to our cohort (our study used the ISRNM criteria and MIS, not SGA).
- Removed a duplicated sentence describing the MIS components and scoring in the Methods.
- Standardized the reporting of very small p-values, replacing the SPSS output “0.000” / “.000” with “<0.001” throughout the tables and text.

In addition, we have moderated two statements about agreement between the two tools. The ISRNM–MIS correlation (r = 0.282) is now described as weak-to-modest rather than moderate, and we note that the two tools identified overlapping but not identical groups of patients and are not interchangeable, given the difference in prevalence (42.5% by ISRNM vs 72.9% by MIS).

We believe these revisions fully address the points raised and have strengthened the manuscript. We thank you and the reviewers again for your careful and constructive assessment.

Yours sincerely,

**Shakil Ahmed, Nasrin Sultana**

on behalf of all authors
