# Supplementary material for: Malnutrition in maintenance hemodialysis: Prevalence, patterns, and clinical implications in Bangladesh
Source: PLoS One. 2026 Jul 17;21(7):e0354173. doi: 10.1371/journal.pone.0354173 (PMC13379082; doi:10.1371/journal.pone.0354173)
Supplement: Peer review history

# Peer review: Decision Letter 0

**DOI:** 10.1371/journal.pone.0354173.r001

**Authors:** Yoshitaka Ishibashi

10 Mar 2026

PONE-D-25-68422

Malnutrition in maintenance hemodialysis: Prevalence, patterns, and clinical implications in Bangladesh

PLOS One

Dear Dr. Ahmed,

Thank you for submitting your manuscript to PLOS ONE. After careful consideration, we feel that it has merit but does not fully meet PLOS ONE’s publication criteria as it currently stands. Therefore, we invite you to submit a revised version of the manuscript that addresses the points raised during the review process.

Please submit your revised manuscript by Apr 22 2026 11:59PM. If you will need more time than this to complete your revisions, please reply to this message or contact the journal office at plosone@plos.org. When you're ready to submit your revision, log on to https://www.editorialmanager.com/pone/ and select the 'Submissions Needing Revision' folder to locate your manuscript file.

Please include the following items when submitting your revised manuscript:

- A letter that responds to each point raised by the academic editor and reviewer(s). You should upload this letter as a separate file labeled 'Response to Reviewers'.
- A marked-up copy of your manuscript that highlights changes made to the original version. You should upload this as a separate file labeled 'Revised Manuscript with Track Changes'.
- An unmarked version of your revised paper without tracked changes. You should upload this as a separate file labeled 'Manuscript'.

If you would like to make changes to your financial disclosure, please include your updated statement in your cover letter. Guidelines for resubmitting your figure files are available below the reviewer comments at the end of this letter.

If applicable, we recommend that you deposit your laboratory protocols in protocols.io to enhance the reproducibility of your results. Protocols.io assigns your protocol its own identifier (DOI) so that it can be cited independently in the future. For instructions see: https://journals.plos.org/plosone/s/submission-guidelines#loc-laboratory-protocols. Additionally, PLOS ONE offers an option for publishing peer-reviewed Lab Protocol articles, which describe protocols hosted on protocols.io. Read more information on sharing protocols at https://plos.org/protocols?utm_medium=editorial-email&utm_source=authorletters&utm_campaign=protocols.

We look forward to receiving your revised manuscript.

Kind regards,

Yoshitaka Ishibashi

Academic Editor

PLOS One

Journal Requirements:

When submitting your revision, we need you to address these additional requirements.

1. Please ensure that your manuscript meets PLOS ONE's style requirements, including those for file naming. The PLOS ONE style templates can be found at

https://journals.plos.org/plosone/s/file?id=wjVg/PLOSOne_formatting_sample_main_body.pdf and https://journals.plos.org/plosone/s/file?id=ba62/PLOSOne_formatting_sample_title_authors_affiliations.pdf

2. Thank you for stating the following in your Competing Interests section:

[NO authors have competing interests].

Please complete your Competing Interests on the online submission form to state any Competing Interests. If you have no competing interests, please state "The authors have declared that no competing interests exist.", as detailed online in our guide for authors at http://journals.plos.org/plosone/s/submit-now

This information should be included in your cover letter; we will change the online submission form on your behalf.

3. We note that your Data Availability Statement is currently as follows: [All relevant data are within the manuscript and its Supporting Information files.]

Please confirm at this time whether or not your submission contains all raw data required to replicate the results of your study. Authors must share the “minimal data set” for their submission. PLOS defines the minimal data set to consist of the data required to replicate all study findings reported in the article, as well as related metadata and methods (https://journals.plos.org/plosone/s/data-availability#loc-minimal-data-set-definition).

For example, authors should submit the following data:

- The values behind the means, standard deviations and other measures reported;

- The values used to build graphs;

- The points extracted from images for analysis.

Authors do not need to submit their entire data set if only a portion of the data was used in the reported study.

If your submission does not contain these data, please either upload them as Supporting Information files or deposit them to a stable, public repository and provide us with the relevant URLs, DOIs, or accession numbers. For a list of recommended repositories, please see https://journals.plos.org/plosone/s/recommended-repositories.

If there are ethical or legal restrictions on sharing a de-identified data set, please explain them in detail (e.g., data contain potentially sensitive information, data are owned by a third-party organization, etc.) and who has imposed them (e.g., an ethics committee). Please also provide contact information for a data access committee, ethics committee, or other institutional body to which data requests may be sent. If data are owned by a third party, please indicate how others may request data access.

If the reviewer comments include a recommendation to cite specific previously published works, please review and evaluate these publications to determine whether they are relevant and should be cited. There is no requirement to cite these works unless the editor has indicated otherwise.

Additional Editor Comments:

Dear Authors,

Thank you for submitting your manuscript entitled

“Malnutrition in maintenance hemodialysis: Prevalence, patterns, and clinical implications in Bangladesh”.

Both reviewers recognize that this study addresses an important and underexplored topic, particularly in resource-limited dialysis settings. The use of both ISRNM criteria and the Malnutrition Inflammation Score (MIS) to assess nutritional status in hemodialysis patients provides potentially valuable clinical insight.

However, several substantial issues were identified during peer review that must be addressed before the manuscript can be considered for publication. Therefore, I am inviting you to submit a revision of your manuscript.

When revising your manuscript, please respond carefully to each of the reviewers’ comments and concerns. In your resubmission, please provide a point-by-pint response to all reviewer comments and clearly indicate how the manuscript has been revised accordingly. If you choose not to follow a particular suggestion, please provide a clear justification.

We look forward to receiving your revised manuscript.

Sincerely,

Academic Editor

Yoshitaka Ishibashi

[Note: HTML markup is below. Please do not edit.]

Reviewers' comments:

Reviewer's Responses to Questions

**Comments to the Author**

1. Is the manuscript technically sound, and do the data support the conclusions?

The manuscript must describe a technically sound piece of scientific research with data that supports the conclusions. Experiments must have been conducted rigorously, with appropriate controls, replication, and sample sizes. The conclusions must be drawn appropriately based on the data presented.

Reviewer #1: Yes

Reviewer #2: Yes

**********

2. Has the statistical analysis been performed appropriately and rigorously?

Reviewer #1: Yes

Reviewer #2: Yes

**********

3. Have the authors made all data underlying the findings in their manuscript fully available?

The PLOS Data policy requires authors to make all data underlying the findings described in their manuscript fully available without restriction, with rare exception (please refer to the Data Availability Statement in the manuscript PDF file). The data should be provided as part of the manuscript or its supporting information, or deposited to a public repository. For example, in addition to summary statistics, the data points behind means, medians and variance measures should be available. If there are restrictions on publicly sharing data—e.g. participant privacy or use of data from a third party—those must be specified.

Reviewer #1: Yes

Reviewer #2: Yes

**********

4. Is the manuscript presented in an intelligible fashion and written in standard English?

PLOS ONE does not copyedit accepted manuscripts, so the language in submitted articles must be clear, correct, and unambiguous. Any typographical or grammatical errors should be corrected at revision, so please note any specific errors here.

Reviewer #1: Yes

Reviewer #2: Yes

**********

5. Review Comments to the Author

Please use the space provided to explain your answers to the questions above. You may also include additional comments for the author, including concerns about dual publication, research ethics, or publication ethics. (Please upload your review as an attachment if it exceeds 20,000 characters)

Reviewer #1: Comments to the author,

The authors showed the clinical characteristics of malnutrition with maintenance HD patients in Bangladesh. This paper is original since no data has yet been presented. I have some comments and concerns regarding this manuscript.

Major points

1. First of all, the number of the study patients was relatively small. In addition, the study design was cross-sectional study. They were a critical limitation of this study.

2. The definition of malnutrition is important in this study. However, I concerned that there is a possibility that its definition is not appropriate in this study. Table 4 shows that the daily energy intake (DEI) and the daily protein intake (DPI) were significantly higher in patients with malnutrition compared to those without malnutrition.

3. The definition of malnutrition was not mentioned in Table 5. Did the authors conduct the validation of the ISRNM and MIS in Bangladesh patients?

4. The authors demonstrated that dialyzer reuse was significantly correlated with malnutrition. I concerned that there is a possibility that the bias may be present. How do the physicians decide to reuse the dialyzer in Bangladesh?

5. In the introduction, the authors mentioned that the prevalence of malnutrition is 42.7% in the world by referring only one study. The universal data should be mentioned on the basis of the several references.

6. The description of MIS components should be mentioned in the manuscript.

7. The results of creatinine and eGFR should be removed in Table because all the study patients undertook maintenance hemodialysis.

Reviewer #2: This paper comprehensively examines the critical and unresolved issue of malnutrition among HD patients in Bangladesh using reliable assessment tools, demonstrating high academic and clinical value. By focusing on the key points mentioned above for revision, it is expected to achieve a level of completeness sufficient for publication in an international journal.

Major Revision

#1 Study design and sampling bias

The use of convenience sampling limits representativeness and external validity.

You may discuss the impact on generalizability and propose probabilistic sampling in future studies.

#2 Limited discussion on discrepancy between ISRNM and MIS

The large difference in malnutrition prevalence between ISRNM and MIS warrants deeper methodological and clinical discussion.

Please clarify differences in sensitivity, subjectivity, and inflammatory components of MIS.

#3 Effect of dialyzer reuse as a predictor

While statistically significant, the biological plausibility is insufficiently explored.

Please discuss the mechanisms such as inflammation, toxin clearance, and infection risk.

Minor Revision

Please recheck some terminology and units' error

Ex.Inconsistent use of “Sr.” vs “Serum” or " μmol//l".

**********

6. PLOS authors have the option to publish the peer review history of their article (what does this mean?). If published, this will include your full peer review and any attached files.

If you choose “no”, your identity will remain anonymous but your review may still be made public.

**Do you want your identity to be public for this peer review?**  For information about this choice, including consent withdrawal, please see our Privacy Policy.

Reviewer #1: No

Reviewer #2: No

**********

[NOTE: If reviewer comments were submitted as an attachment file, they will be attached to this email and accessible via the submission site. Please log into your account, locate the manuscript record, and check for the action link "View Attachments". If this link does not appear, there are no attachment files.]

To ensure your figures meet our technical requirements, please review our figure guidelines: https://journals.plos.org/plosone/s/figures

You may also use PLOS’s free figure tool, NAAS, to help you prepare publication quality figures: https://journals.plos.org/plosone/s/figures#loc-tools-for-figure-preparation.

NAAS will assess whether your figures meet our technical requirements by comparing each figure against our figure specifications.

# Author comment: Author response to Decision Letter 1

**DOI:** 10.1371/journal.pone.0354173.r002

20 Apr 2026

Response to Reviewer 1:

1. The authors showed the clinical characteristics of malnutrition with maintenance HD patients in Bangladesh. This paper is original since no data has yet been presented. I have some comments and concerns regarding this manuscript.

Answer: We sincerely thank the reviewer for the insightful and constructive comments. We have carefully revised the manuscript to address all concerns. Our detailed responses are provided below.

2. First of all, the number of the study patients was relatively small. In addition, the study design was cross-sectional study. They were a critical limitation of this study.

Answer: Thank you for this insightful comment. We acknowledge that the relatively small sample size is a limitation of this study. The smaller number of participants was mainly due to practical challenges, including limited willingness of patients to participate in research surveys, restricted access to dialysis centers, incomplete medical record documentation, and the need for offline, face-to-face data collection during dialysis sessions. We have now clarified this point in the “Strengths and Limitations” section. In addition, we have cited comparable state-of-the-art studies [25, 33, 45, 48] with similar or less sample sizes.

Regarding study design, the aim of this study was to assess the prevalence of malnutrition among hemodialysis patients in a resource-limited setting in Bangladesh. Therefore, a cross-sectional design is appropriate for generating primary data and estimating disease prevalence, as it provides a snapshot of a specific population at a single point in time in a relatively fast and cost-effective manner.

3. The definition of malnutrition is important in this study. However, I concerned that there is a possibility that its definition is not appropriate in this study. Table 4 shows that the daily energy intake (DEI) and the daily protein intake (DPI) were significantly higher in patients with malnutrition compared to those without malnutrition.

Answer: We appreciate this important observation and thank you for your comment. In this study, malnutrition was defined using established ISRNM criteria and the MIS, which incorporate anthropometric, biochemical, clinical, and dietary indicators rather than relying solely on dietary intake. Therefore, we have revised the Results and Discussion sections to clarify the interpretation of Table 4.

Additionally, we would like to acknowledge that we initially applied an incorrect cut-off point for under-reporting Goldberg Index = (Energy Intake / Basal Metabolic Rate) using >0.9. However, for hemodialysis (HD) patients, a study used cut-off is 0.76 [35]. We have now corrected this and recalculated the data accordingly, and the revised results have been updated in the manuscript.

4. The authors demonstrated that dialyzer reuse was significantly correlated with malnutrition. I concerned that there is a possibility that the bias may be present. How do the physicians decide to reuse the dialyzer in Bangladesh?

Answer: Thank you for this important observation. In Bangladesh, dialyzer reuse is primarily influenced by economic constraints and institutional practices rather than patient nutritional status. Patients often reuse dialyzers to reduce the financial burden of repeated dialysis sessions. Therefore, dialyzer reuse may also reflect underlying socioeconomic conditions that are associated with increased risk of malnutrition. We have clarified this point in the Discussion section and acknowledged the possibility of residual confounding.

5. In the introduction, the authors mentioned that the prevalence of malnutrition is 42.7% in the world by referring only one study. The universal data should be mentioned on the basis of the several references.

Answer: Thank you for this valuable suggestion. We have revised the Introduction to reflect variability in global prevalence of malnutrition among hemodialysis patients by citing multiple references rather than relying on a single estimate.

6. The description of MIS components should be mentioned in the manuscript.

Answer: Thank you for your insightful comment. We have expanded the Methods section to clearly describe the 10 components of the MIS scoring system.

7. The results of creatinine and eGFR should be removed in Table because all the study patients undertook maintenance hemodialysis.

Answer: Thank you for this valuable comment. We agree that creatinine and eGFR are not clinically informative parameters in maintenance hemodialysis patients. Therefore, we have removed these variables from Table 1 and Table 3 and revised the corresponding text accordingly.

Response to Reviewer 2:

1. This paper comprehensively examines the critical and unresolved issue of malnutrition among HD patients in Bangladesh using reliable assessment tools, demonstrating high academic and clinical value. By focusing on the key points mentioned above for revision, it is expected to achieve a level of completeness sufficient for publication in an international journal.

Answer: We thank the reviewer for the valuable suggestions, which have significantly improved the manuscript.

2. Study design and sampling bias. The use of convenience sampling limits representativeness and external validity. You may discuss the impact on generalizability and propose probabilistic sampling in future studies.

Answer: Thank you for this valuable comment. We have now clarified in the Strengths and Limitations section that convenience sampling and recruitment from a limited number of dialysis centers may affect representativeness of the findings. We also highlighted that future studies using larger, multicenter samples and probabilistic sampling approaches are recommended to improve robustness and representativeness.

3. Limited discussion on discrepancy between ISRNM and MIS. The large difference in malnutrition prevalence between ISRNM and MIS warrants deeper methodological and clinical discussion. Please clarify differences in sensitivity, subjectivity, and inflammatory components of MIS.

Answer: Thank you for this important suggestion. We have expanded the Discussion section to clarify the discrepancy in malnutrition prevalence between ISRNM and MIS. MIS incorporates both objective biochemical parameters and subjective clinical components, including factors related to inflammation, functional status, and comorbidity burden, which can increase its sensitivity in detecting early or subclinical protein-energy wasting. In contrast, ISRNM relies primarily on objective criteria across defined nutritional domains. These methodological differences are explained the higher prevalence of malnutrition identified by MIS in our study.

4. Effect of dialyzer reuse as a predictor. While statistically significant, the biological plausibility is insufficiently explored. Please discuss the mechanisms such as inflammation, toxin clearance, and infection risk.

Answer: Thank you for this valuable comment. We have expanded the Discussion section to further explore the biological plausibility of the association between dialyzer reuse and malnutrition. Repeated use of dialyzers can reduce membrane efficiency, potentially leading to inadequate toxin clearance, while exposure to bioincompatible materials can contribute to chronic inflammation. In addition, reuse practices can increase infection risk. These clinical factors, together with underlying economic constraints influencing reuse practices, can contribute to increased risk of protein-energy wasting among hemodialysis patients. The relevant discussion has been added to improve interpretation of this finding.

5. Minor Revision. Please recheck some terminology and units' error. Ex.Inconsistent use of “Sr.” vs “Serum” or " μmol//l".

Answer: Thank you for pointing this out. We have carefully reviewed the manuscript and corrected inconsistencies in terminology and unit formatting throughout the text, tables, and figures. In particular, abbreviations such as “Sr.” were replaced with the standardized term “Serum,” and unit formatting (e.g., μmol/L, mg/dL, kg/m²) was revised for consistency according to journal style. In the table we used Sr. shortform and its full form is mentioned in every table footnote.

Attachment Submitted filename: Response to Reviewers.docx

# Editor report: Decision Letter 1

**DOI:** 10.1371/journal.pone.0354173.r003

**Authors:** Yoshitaka Ishibashi

8 May 2026

PONE-D-25-68422R1

Malnutrition in maintenance hemodialysis: Prevalence, patterns, and clinical implications in Bangladesh

PLOS One

Dear Dr. Ahmed,

Thank you for submitting your manuscript to PLOS ONE. After careful consideration, we feel that it has merit but does not fully meet PLOS ONE’s publication criteria as it currently stands. Therefore, we invite you to submit a revised version of the manuscript that addresses the points raised during the review process.

==============================

**ACADEMIC EDITOR:**

I have carefully evaluated your revised manuscript and your detailed responses to the reviewers. The reviewers raised several important concerns regarding: the definition and assessment of malnutrition, the discrepancy between ISRNM and MIS, the interpretation of dietary intake findings, and the potential bias and interpretation of dialyzer reuse. I appreciate that you have made substantial efforts to address these issues. In particular, the clarification of malnutrition criteria, the correction of the Goldberg index cut-off, and the expanded discussion on methodological differences and biological plausibility have improved the manuscript. However, a few points still require clarification before the manuscript can be considered for publication:

1. Interpretation of dietary intake findings. Although revised, the apparent inconsistency between higher intake and malnutrition status should be explained more clearly and cautiously.
2. Dialyzer reuse and confounding. While socioeconomic context is mentioned, the potential residual confounding and causal interpretation should be further toned down.
3. Methodological limitations. The implications of convenience sampling and cross-sectional design on generalizability should be emphasized more explicitly.
4. Clarity and consistency. Minor issues in terminology, unit presentation, and clarity of tables should be carefully rechecked.

With revisions addressing the points above, we will re-consider the manuscript. I look forward to receiving your revised manuscript.

==============================

Please submit your revised manuscript by Jun 22 2026 11:59PM. If you will need more time than this to complete your revisions, please reply to this message or contact the journal office at plosone@plos.org. When you're ready to submit your revision, log on to https://www.editorialmanager.com/pone/ and select the 'Submissions Needing Revision' folder to locate your manuscript file.

Please include the following items when submitting your revised manuscript:

- A letter that responds to each point raised by the academic editor and reviewer(s). You should upload this letter as a separate file labeled 'Response to Reviewers'.
- A marked-up copy of your manuscript that highlights changes made to the original version. You should upload this as a separate file labeled 'Revised Manuscript with Track Changes'.
- An unmarked version of your revised paper without tracked changes. You should upload this as a separate file labeled 'Manuscript'.

If you would like to make changes to your financial disclosure, please include your updated statement in your cover letter. Guidelines for resubmitting your figure files are available below the reviewer comments at the end of this letter.

If applicable, we recommend that you deposit your laboratory protocols in protocols.io to enhance the reproducibility of your results. Protocols.io assigns your protocol its own identifier (DOI) so that it can be cited independently in the future. For instructions see: https://journals.plos.org/plosone/s/submission-guidelines#loc-laboratory-protocols. Additionally, PLOS ONE offers an option for publishing peer-reviewed Lab Protocol articles, which describe protocols hosted on protocols.io. Read more information on sharing protocols at https://plos.org/protocols?utm_medium=editorial-email&utm_source=authorletters&utm_campaign=protocols.

As the corresponding author, your ORCID iD is verified in the submission system and will appear in the published article. PLOS supports the use of ORCID, and we encourage all coauthors to register for an ORCID iD and use it as well. Please encourage your coauthors to verify their ORCID iD within the submission system before final acceptance, as unverified ORCID iDs will not appear in the published article. *Only*  the individual author can complete the verification step; PLOS staff *cannot*  verify ORCID iDs on behalf of authors.

We look forward to receiving your revised manuscript.

Kind regards,

Yoshitaka Ishibashi

Academic Editor

PLOS One

**Journal Requirements:**

If the reviewer comments include a recommendation to cite specific previously published works, please review and evaluate these publications to determine whether they are relevant and should be cited. There is no requirement to cite these works unless the editor has indicated otherwise.

**Additional Editor Comments:**

In light of the two reviewer's comment, major revision would be necessary before it can be considered for acceptance.

The topic is important and the dataset is potentially valuable, but further refinement—particularly in methodological interpretation and discussion—is necessary to meet the journal’s standards.

I encourage the authors to address all comments carefully and provide a clear, point-by-point response in the next revision.

Sincerely,

Yoshitaka Ishibashi, MD, PhD

Academic Editor, PLOS ONE

[Note: HTML markup is below. Please do not edit.]

[NOTE: If reviewer comments were submitted as an attachment file, they will be attached to this email and accessible via the submission site. Please log into your account, locate the manuscript record, and check for the action link "View Attachments". If this link does not appear, there are no attachment files.]

To ensure your figures meet our technical requirements, please review our figure guidelines: https://journals.plos.org/plosone/s/figures

You may also use PLOS’s free figure tool, NAAS, to help you prepare publication quality figures: https://journals.plos.org/plosone/s/figures#loc-tools-for-figure-preparation.

NAAS will assess whether your figures meet our technical requirements by comparing each figure against our figure specifications.

# Author comment: Author response to Decision Letter 2

**DOI:** 10.1371/journal.pone.0354173.r004

22 Jun 2026

1. Interpretation of dietary intake findings

“Although revised, the apparent inconsistency between higher intake and malnutrition status should be explained more clearly and cautiously.”

Response: We are grateful for this observation. On re-examining our data we found that the earlier wording was inaccurate: malnourished patients did not have higher intake. As shown in Table 4, malnourished patients had significantly lower absolute energy and protein intakes than non-malnourished patients. However, energy intake expressed per kilogram of body weight (DEI) did not differ significantly between the groups (approximately 26–27 kcal/kg/day) and approached recommended levels. We have rewritten the relevant paragraph in the Discussion to state this accurately and cautiously, and to explain that malnutrition in hemodialysis is multifactorial and may occur despite seemingly adequate weight-adjusted intake owing to inflammation, metabolic and hormonal disturbances, and dialysis-related nutrient losses. We also clarify that nutritional status was interpreted using combined anthropometric, biochemical, and clinical indicators rather than dietary intake alone.

2. Dialyzer reuse and confounding

“While socioeconomic context is mentioned, the potential residual confounding and causal interpretation should be further toned down.”

Response: We have toned down the causal interpretation in the Discussion. The text now states explicitly that, because dialyzer reuse is closely tied to socioeconomic status in this setting, the observed association is likely subject to residual confounding and that a causal relationship cannot be inferred from these cross-sectional data. We have added that the estimate is statistically imprecise: only 19 patients reused dialyzers more than twice, producing a wide confidence interval (AOR = 5.8; 95% CI: 1.2–28.7). The finding is now described as hypothesis-generating, and we note that prospective studies measuring and adjusting for socioeconomic and inflammatory factors are needed. In addition, the proposed biological mechanisms (reduced membrane efficiency, exposure to bio-incompatible materials, inflammation, and infection risk) are now explicitly framed as hypothesized pathways that were not measured in the present study.

3. Methodological limitations

“The implications of convenience sampling and cross-sectional design on generalizability should be emphasized more explicitly.”

Response: We have expanded the Strengths and Limitations section. We now state that, because convenience sampling was used, the cohort is likely to over-represent more stable and treatment-adherent patients attending established urban dialysis centers, while under-representing those in primary or rural facilities, those not currently receiving dialysis, and those who declined participation; the direction and magnitude of this selection bias cannot be quantified. We state explicitly that the prevalence estimates and associations reported here should be interpreted as specific to the studied centers and should not be generalized to the national maintenance hemodialysis population. We further note that, combined with the cross-sectional design, these features mean the results describe associations at a single time point rather than causal or temporal relationships.

4. Clarity and consistency

“Minor issues in terminology, unit presentation, and clarity of tables should be carefully rechecked.”

Response: We have rechecked terminology, units, and tables throughout, and made the following corrections (all shown as tracked changes):

• Corrected a statement that “ISRNM identified 58% of patients as malnourished” (before Table 3); 58% was the proportion with low BMI (<23 kg/m²). The ISRNM malnutrition prevalence is 42.5% (meeting at least three of the four domains), and the text now reads accordingly.

• Reconciled the dietary-inadequacy percentages in the Discussion (previously stated as 47% and 20.4%) with Table 2, which reports 42.5% for energy intake <25 kcal/kg/day and 31.5% for protein intake <0.8 g/kg/day.

• Clarified that the figure of “more than 40% malnourished by SGA” refers to the cited Jordanian study, not to our cohort (our study used the ISRNM criteria and MIS, not SGA).

• Removed a duplicated sentence describing the MIS components and scoring in the Methods.

• Standardized the reporting of very small p-values, replacing the SPSS output “0.000” / “.000” with “<0.001” throughout the tables and text.

In addition, we have moderated two statements about agreement between the two tools. The ISRNM–MIS correlation (r = 0.282) is now described as weak-to-modest rather than moderate, and we note that the two tools identified overlapping but not identical groups of patients and are not interchangeable, given the difference in prevalence (42.5% by ISRNM vs 72.9% by MIS).

We believe these revisions fully address the points raised and have strengthened the manuscript. We thank you and the reviewers again for your careful and constructive assessment.

Yours sincerely,

Shakil Ahmed, Nasrin Sultana

on behalf of all authors

Attachment Submitted filename: Response_to_Reviewers_auresp_2.docx

# Editor report: Decision Letter 2

**DOI:** 10.1371/journal.pone.0354173.r005

**Authors:** Yoshitaka Ishibashi

5 Jul 2026

Malnutrition in maintenance hemodialysis: Prevalence, patterns, and clinical implications in Bangladesh

PONE-D-25-68422R2

Dear Dr. Ahmed,

We’re pleased to inform you that your manuscript has been judged scientifically suitable for publication and will be formally accepted for publication once it meets all outstanding technical requirements.

Within one week, you’ll receive an e-mail detailing the required amendments. When these have been addressed, you’ll receive a formal acceptance letter and your manuscript will be scheduled for publication.

An invoice will be generated when your article is formally accepted. Please note, if your institution has a publishing partnership with PLOS and your article meets the relevant criteria, all or part of your publication costs will be covered. Please make sure your user information is up-to-date by logging into Editorial Manager at Editorial Manager® and clicking the ‘Update My Information' link at the top of the page. For questions related to billing, please contact billing support.

If your institution or institutions have a press office, please notify them about your upcoming paper to help maximize its impact. If they’ll be preparing press materials, please inform our press team as soon as possible -- no later than 48 hours after receiving the formal acceptance. Your manuscript will remain under strict press embargo until 2 pm Eastern Time on the date of publication. For more information, please contact onepress@plos.org.

Kind regards,

Yoshitaka Ishibashi

Academic Editor

PLOS One

# Editor report: Acceptance letter

**DOI:** 10.1371/journal.pone.0354173.r006

**Authors:** Yoshitaka Ishibashi

PONE-D-25-68422R2

PLOS One

Dear Dr. Ahmed,

I'm pleased to inform you that your manuscript has been deemed suitable for publication in PLOS One. Congratulations! Your manuscript is now being handed over to our production team.

At this stage, our production department will prepare your paper for publication. This includes ensuring the following:

* All references, tables, and figures are properly cited

* All relevant supporting information is included in the manuscript submission,

* There are no issues that prevent the paper from being properly typeset

You will receive further instructions from the production team, including instructions on how to review your proof when it is ready. Please keep in mind that we are working through a large volume of accepted articles, so please give us a few days to review your paper and let you know the next and final steps.

Lastly, if your institution or institutions have a press office, please let them know about your upcoming paper now to help maximize its impact. If they'll be preparing press materials, please inform our press team within the next 48 hours. Your manuscript will remain under strict press embargo until 2 pm Eastern Time on the date of publication. For more information, please contact onepress@plos.org.

You will receive an invoice from PLOS for your publication fee after your manuscript has reached the completed accept phase. If you receive an email requesting payment before acceptance or for any other service, this may be a phishing scheme. Learn how to identify phishing emails and protect your accounts at https://explore.plos.org/phishing.

If we can help with anything else, please email us at customercare@plos.org.

Thank you for submitting your work to PLOS ONE and supporting open access.

Kind regards,

PLOS ONE Editorial Office Staff

on behalf of

Dr. Yoshitaka Ishibashi

Academic Editor

PLOS One
